# Supplementary material for: The proportion of Thai postmenopausal women who would be eligible for anti-osteoporosis therapy
Source: PLoS One. 2023 Feb 3;18(2):e0279829. doi: 10.1371/journal.pone.0279829 (PMC9897565; doi:10.1371/journal.pone.0279829)
Supplement: Attachment — Submitted filename: Response to reviewer PLOS NP CP.docx [file pone.0279829.s002.docx]

Dear Editor Board Member,

PLOS ONE

Thank you very much for your letter dated September 5, 2022 together with the reviewer’s comments our manuscript. We very much appreciate the thoughtful and helpful review of our work, and thank the reviewer for the suggestions to enhance the manuscript. We have carefully considered the comments and would like to take the opportunity to address to the comments in the following attachment. Please see the attached for our point-by-point response. Changes are highlighted in red in the revised manuscript.

We consider that the manuscript has improved significantly as a result of the reviewer’s comments. We hope that the revised manuscript is satisfactory to you, and is possible for publication in PLOS ONE.

Your Sincerely,

**Chatlert Pongchaiyakul**

Division of Endocrinology and Metabolism

Department of Medicine, Faculty of Medicine

Khon Kaen University, THAILAND 40002

Tel. +6643363664	FAX: +6643202491

Email: pchatl@kku.ac.th

Corresponding author

**RESPONSE TO REVIEWERS**

**Reviewer #1**

The strength of study is large sample size. The manuscript is overall well written and informative. The study was properly designed and executed. The finding can help inform treatment guidelines in Thailand. However, there are some issues that I would like to invite the authors to comment on.

Response: We would like to thank the reviewer for the helpful and thoughtful review of our manuscript.

1. Please clarify the define of postmenopausal in this study? How many women aged 40-50 were there? Did all of them have natural or premature/induced menopause?

Response: Thank you for the suggestion, we have included the definition of postmenopausal in the study in the methods section. We excluded patients with medication-induced or surgical menopause (Lines 79). We also included number of women in each age groups in the results section (Lines 118 – 119).

2. In the methods, please describe more details about prior fractures (trauma or non-trauma)? Response: Thank you for the suggestion, we have included additional description on ascertainment of prior fracture (Lines 83).

3. The point estimate of the proportion of Thai menopausal women who would be eligible for treatment was given (37.3%). Please provide the related confidence intervals, quantifying the potential uncertainty of the finding which would make the interpretation stronger and more convincingly.

Response: Thank you for the suggestion, we have included the 95% CI of the effect size in the manuscript (Lines 169 and 180).

4. The authors defined “high-risk osteopenia” (as participants with osteopenia and 10-year probability of hip fractures with BMD ≥ 3% and/or MOF with BMD ≥ 20%.), but I think the term “high risk of fracture” is more appropriate.

Response: We have made the recommended change throughout the manuscript.

5. Please discuss more about the likelihood of vertebral fracture was not significantly difference between participants with and without osteoporosis

Response: Thank you for the recommendation, we have added discussion on the potential explanation of the observation in the manuscript (Lines 215 – 219).
